# Community Engagement and Outreach Programs for Lead Prevention in Mississippi

**DOI:** 10.3390/ijerph18010202

**Published:** 2020-12-29

**Authors:** Amal K. Mitra, Charkarra Anderson-Lewis

**Affiliations:** 1 School of Public Health, College of Health Sciences, Jackson State University, Jackson, MS 39213, USA; 2 School of Health Professions, The University of Southern Mississippi, Hattiesburg, MS 39406, USA; charkarra.andersonlewis@usm.edu

**Keywords:** lead poisoning, children, prevention, outreach, health education, sources, complications, Mississippi

## Abstract

The objective of the project was to encourage health promotion through education, outreach, and community-based training. The people attending health fairs (*n* = 467), community events (*n* = 469), and Kindergarten classes (*n* = 241) were the study participants. Hands-on training was offered at homebuilding retail stores (*n* = 25). U.S. Department of Housing and Urban Development (HUD)’s online visual training was given to realtors (*n* = 220), and inspectors, contractors, and Do-It-Yourself (DIY) workers (*n* = 75). Training workshops were attended by home-buyers and rental home owners at the Neighborhood Association Meetings (*n* = 91). The impact of training was evaluated by pre- and posttests. Nearly, 90% of the participants (*n* = 25) reported the hands-on training was useful. At posttest after the HUD online training, 59.4%, 67.9%, 65.1% of the participants (*n* = 220) identified soil, car batteries, and paint as sources of lead in the environment, respectively. Nearly 70% identified lead as a poison in the environment while 77.5% and 47.2% demonstrated two behaviors which help prevent lead poisoning. A total of 62.3%, 48.1%, and 58.5%, at posttest identified three complications or illnesses—behavioral, physical, and psychological, respectively. The home owners are required to get permission from the City for housing repair. In coordination with the federally funded housing repair or lead abatement programs, the trained inspectors are authorized to certify the renovation or repair works. These outreach activities were successful in improving the knowledge of the community people on lead poisoning prevention.

## 1. Introduction

Globally, 23% of all deaths and 26% of deaths among children under age 5 are due to preventable environmental factors [1]. The Environmental Protection Agency recognizes lead poisoning as one of the most preventable health tragedies for children in the United States [2]. Unfortunately, some populations and geographic areas in the U.S. remain at disproportionately high risk for lead exposure [3]. According to the U.S. Department of Urban and Housing Development, there are approximately 3.8 million houses or buildings that have children living in them who are potentially being exposed to lead [4]. Children of some racial and ethnic groups, such as non-Hispanic African-Americans, are at higher risk for exposure to lead. Nearly half a million U.S. children ages 1 to 5 have blood lead levels at or above 5 µg/dL, which is currently the reference level at which the Centers for Disease Control and Prevention (CDC) recommends public health actions be taken [5].

Lead toxicity affects almost every organ system of the human body [6]. Long-term exposure to lead can seriously harm a child’s health and cause well-documented adverse effects including neurological damage [7], retarded growth and development [8], learning and behavioral abnormalities [9,10], hearing and speech problems [10,11], deficits in cognitive function [12], sleep deficits [13], attention deficiencies, and underperformance in school [13].

The Healthy People 2020 [14] and the proposed Healthy People 2030 objectives [15] established the nation’s strategy for improving the health and well-being of all citizens, and emphasized to reduce blood lead level in children aged 1–5 years. According to CDC, there is an urgent need to develop health education and outreach activities that are culturally appropriate and sensitive to the target population in reducing disparities in lead poisoning [3].

To provide a comprehensive effort to educating community people on childhood exposure to lead and lead prevention, we developed a community-based participatory research (CBPR) program called Community Lead Awareness Partnership (CLAP) for Healthy Kids in Mississippi. The aims of the program were three-fold: (1) Identify the most affected communities and the risk group of people having high blood level of lead in Mississippi; (2) Evaluate effectiveness of a comprehensive lead education and training program in awareness building and practices on lead prevention among the most affected populations in Mississippi; and (3) Encourage sustainable infrastructure development programs for the prevention of childhood lead poisoning.

## 2. Materials and Methods

### 2.1. Community Partnership

**Figure 1:**
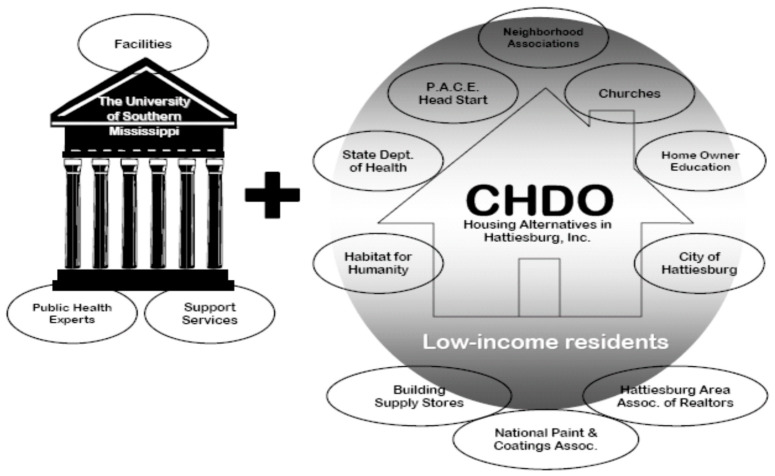
A list of community partners involved in the program.

In this CBPR program, we developed partnership with a number of community organizations and stakeholders in Mississippi (Figure 1). The partners included Mississippi State Department of Health Lead Poisoning Prevention and Healthy Home Program (LPPHHP), the National Paint and Coatings Association (NPCA), a local Community Housing Development Organization (CHDO), Hattiesburg City Government, the Head Start Program, community and faith-based organizations, day-care centers, public schools, home buyers, local contractors, and realtors. The program objectives and activities were developed based on discussions with the partners. A Community Advisory Board (CAB) comprising of representatives from each partner organization and the investigators met quarterly to discuss on strategies and monitor progresses.

### 2.2. Identification of High-Risk Populations

**Figure 2:**
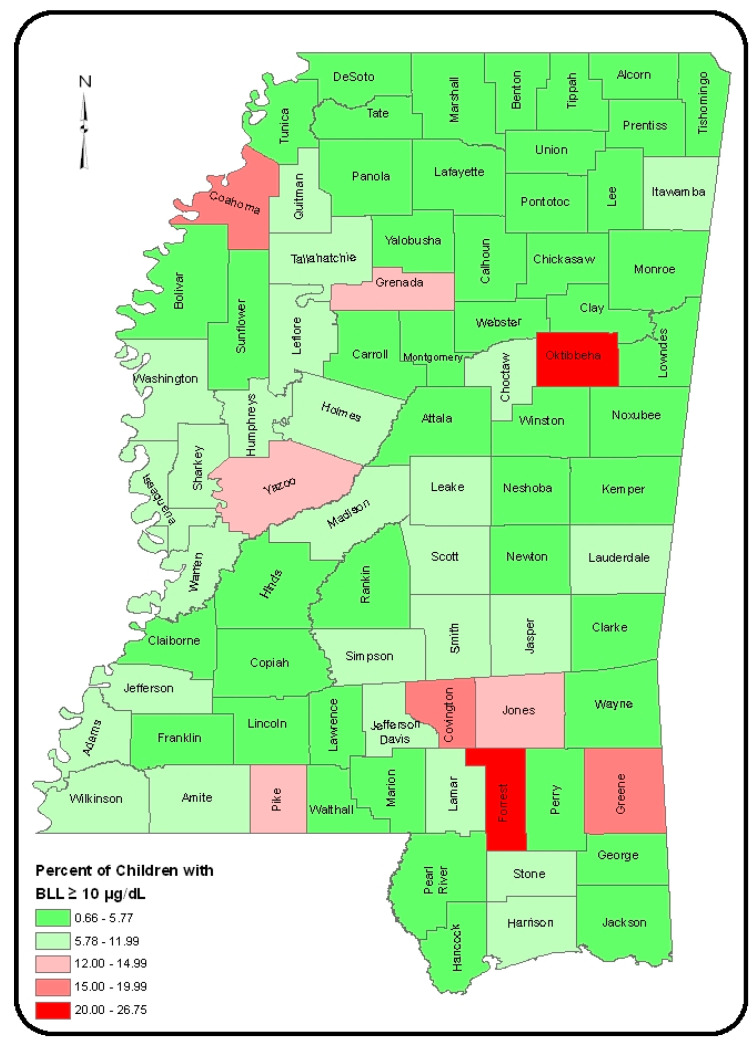
Percent of children with blood levels of lead in 82 countries of Mississippi.

We used the CDC criteria for the identification of “high risk” areas, which states that if 12% or more of children tested for blood lead levels (BLL) are found with raised levels of lead, the area will be designated as a “high-risk” area [16]. In order to focus our efforts in the most vulnerable populations with lead poisoning, we analyzed 42,372 records of children aged less than 6 years in Mississippi. The data were obtained from the Mississippi State Department of Health LPPHHP. Overall, 2446 children in Mississippi (5.8%) had high blood lead levels (BLL) ≥ 5 µg/dL. However, nine counties in the state reported 12% or more of the children tested had high BLL. These counties, in order of the highest to the lowest levels of BLL included Forrest, Oktibbeha, Covington, Coahoma, Greene, Grenada, Pike, Jones, and Yazoo (Figure 2). The City of Hattiesburg, being the largest city in the Forrest County of Mississippi, and having the highest proportion of childhood BLL, was selected as the study site. The project activities targeted population living in the areas of low-income residences of Forrest County.

Results of the secondary data analysis of the Mississippi State Department of Health (MSDH) data and the impact of the lead education and training programs are presented in Figure 2.

### 2.3. Project Goals, Activities, and Measurable Outcomes

Goal 1. Encourage health promotion by conducting community-based outreach concerning childhood lead poisoning prevention.

Activity 1. Provide health education materials, educational training, and presentations to community-based organizations, child-care centers, and faith-based communities. Specific project output: Participate in 10 health fairs; disseminate 600 educational materials; and make presentations to 10 local community-based partner organizations. Measurable outcomes: At post-test, 90% of these participants are able to (a) Identify three primary sources of lead in the environment (paint, dust, and soil); (b) Identify lead as a poison found in the environment and demonstrate a minimum of two behaviors which will help prevent lead poisoning; and (c) Identify a minimum of three complications (physical, mental, or psychological) of lead poisoning.

Activity 2. Partner with local home-builder retail stores (such as Home Depot and Lowe’s) to host hands-on training on lead poisoning prevention. Specific project output: Conduct two training sessions on lead poisoning prevention and lead-safe home repairs (one in each facility). Measurable outcomes: 50% reported the hands-on training was useful for prevention from lead contamination.

Activity 3. Encourage all realtors to take the U.S. Department of Housing and Urban Development (HUD)’s free online Lead-Based Paint Visual Assessment Training [17]. Specific project output: 75 Hattiesburg realtors will complete the online training. Measurable outcomes: 90% of participants report they will use information obtained in the training.

Goal 2. Encourage health promotion by conducting community-based training activities on childhood lead poisoning prevention.

Activity 1. Conduct home-buyer education classes that include curriculum on lead poisoning prevention. Specific project output: Conduct 12 home-buyer education classes that include curriculum on lead poisoning prevention; and yearly home maintenance education course. Measurable outcomes: At post-test, 75% of these participants are able to (a) Identify three primary sources of lead in the environment (paint, dust, and soil); (b) Identify lead as a poison found in the environment and demonstrate a minimum of two behaviors which will help prevent lead poisoning; and (c) Identify a minimum of three complications (physical, mental, or psychological) of lead poisoning.

Activity 2. Provide intensive training on lead-safe work practices to inspectors, contractors, and Do-It-Yourself (DIY) workers. Specific project output: Train 3 inspectors, 25 contractors, and 25 DIY workers on the 8-h U.S. Department of Housing and Urban Development (HUD) curriculum on lead. Measurable outcomes: 75% of participants will report at follow-up survey that they use information obtained during the training.

Activity 3. Provide training in lead-safe work practices and compliance with Toxic Substances Control Act (TSCA) Section 1018 to rental property owners. Specific project output: Hold one seminar for rental property owners on lead-safe work practices. Measurable outcomes: At follow-up, 60% of participants will report they use the information presented at the seminar.

### 2.4. Data Collection Instruments

A summary of the training activities, data collection procedures, training materials, and the assessment methods are presented in Table 1. The assessment instruments were: (1) Exit survey for participants for hands-on training at home builder retail stores—10 questions on a scale from 0 to 10; (2) Follow-up survey for HUD’s Online Training for Realtors—6 questions on a scale from 0 to 10; (3) Pre- and posttest of training of home buyers—5 questions; and (4) HUD Curriculum for Inspectors, Contractors, DIY Workers—8 questions.

## 3. Results

The CLAP for Healthy Kids project staff distributed educational materials and presented lectures on lead prevention at several venues and making over 50 public appearances. These events included health fairs, neighborhood meetings, community events, and events at local parks and schools. The total number of participants in this study were 1588 including those who participated in health fairs (*n* = 467), community events (*n* = 469), and Kindergarten classes (*n* = 241), hands-on training at homebuilding retail stores (*n* = 25), HUD’s online training (*n* = 295), and training workshops for home-buyers and rental home owners (*n* = 91) (Table 2).

### Impact of Hands-On Training, HUD Online Training, and Workshops

Hands-on training was offered at homebuilding retail stores such as Lowe’s, Home Depot, Marvin’s, and Sherwin Williams. Of 25 participants, 23 (92%) reported the hands-on training was very useful or useful on a scale from 1 to 10. HUD’s online Lead-Based Paint Visual Assessment Training was given to 220 realtors, and 75 inspectors, contractors, and DIY workers. Training workshops were attended by 91 home-buyers and rental home owners at the Neighborhood Association Meetings.

At posttest, 59.4%, 67.9%, 65.1% of the realtors (*n* = 220) identified soil, car batteries and paint as sources of lead in the environment, respectively. Nearly 70% identified lead as a poison in the environment while 77.5% and 47.2% of those surveyed demonstrated two different behaviors which will help prevent lead poisoning. A total of 62.3%, 48.1%, and 58.5%, at posttest, identified three complications—behavioral, physical, and psychological, respectively. The mean posttest score was significantly higher than the pretest scores (7.47 ± 2.07 *vs*. 6.60 ± 1.68, *p* = 0.04, respectively).

All the participants on HUD online training (*n* = 75) who participated at a 2-month follow-up survey reported that they actually implemented what they had learned during the training on HUD curriculum on lead. The outcome measurements of home-buyer workshops were not significantly different from those of the online training.

## 4. Discussion

Through the CLAP for Healthy Kids project on lead prevention in Mississippi, a total of 1588 participants took the services. The project offered a number of outreach activities among a wide range of people through health fairs and community events, kindergarten school-based training, training in faith-based organizations and churches, HUD’s online free Lead-Based Paint Visual Assessment Training [17], hands-on training, workshops, and lectures. The impact of outreach programs and trainings were successful in improving knowledge of the community people on lead poisoning prevention.

For certain programs such as tenant-based Section 8 rental assistance, the HUD Regulation on Lead-Based Paint Hazards in Federally Owned Housing and Housing Receiving Federal Assistance requires: (1) Visual assessment of housing units for deteriorated paint; (2) The stabilization and repair of all deteriorated paint; and (3) The visual assessor must be trained to perform a visual assessment [20]. Environmental Protection Agency (EPA)’s Renovation, Repair, and Painting (RRP) Rule requires that firms performing renovation, repair, and painting projects use certified renovators who are trained by EPA-approved training providers and follow lead-safe work practices [21]. The training programs of this project were, therefore, in alignment with the EPA/HUDS’s objective of encouraging sustainable infrastructure development programs for the prevention of childhood lead poisoning.

Among the programs offered by the project, the number of participants at home-building retail stores were not satisfactory. This is a lesson learned that the training programs offered through retail stores such as Lowe’s and Home Depot may not be suitable because of people’s busy schedules and limited time in spending for hands-on training during shopping. One of the most successful of all programs was the EPA/HUD’s online free Lead-Based Paint Visual Assessment Training [17]. The reason of success of this program was probably because people had flexibility of scheduling their time for the online training. It is noteworthy that the outcome measurements of home-buyer face-to-face workshops were not significantly different from those of the online training.

One of the strengths of this study was the community engagement from the beginning of the study in the planning, goal setting, project activities, and project evaluation. The project activities were boost up by the proclamation of the Honorable Mayor of The City of Hattiesburg. CBPR and engagement of the community people have been emphasized in many studies for the success of a community-based program [22]. The project also helped in the sustainability of the lead prevention program in the City by providing training of the home inspectors, DIY workers, realtors, home buyers, and the general people.

Similar success stories of educational programs were reported from another community-participatory research in Philadelphia involving 1200 children and 900 adults [23]. In the later study, community-developed strategies were created for this project with resident leaders from the community and grassroots agencies serving the community. The grassroots agencies included the Philadelphia Housing Authority Tenant Councils for Norris Homes and Apartments and Fairhill Apartments; the Village of the Arts and Humanities, an organization devoted to introducing the arts and humanities to all socioeconomic groups; the Philadelphia Parent Child Center; the Neighborhood Action Bureau, an economic development corporation; and the Salvation Army.

Another community-based Tribal Efforts Against Lead (TEAL) Project used a lay health advisor model to build capacity in a Native American community to reduce lead exposure in a mining area in northeastern Oklahoma [24]. In the TEAL project, approximately 40 tribal members were recruited from area tribes and trained on lead poisoning and its prevention. For a 2-year period, they educated members of their social networks and worked to implement change in their community to reduce exposure to lead.

One of the limitations of the present study was that it focused on health education only. Although several studies have shown successes in improving education of the people, education alone has a limited effectiveness in alleviating the burden of lead poisoning, especially if it is not combined with resources to actually correct lead-based paint hazards in housing or take remedial measures for other sources of lead poisoning. Studies that evaluated the effectiveness of parents’ education alone have failed to show significant reductions in childhood BLLs [25]. Studies are needed to focus on reducing the sources of childhood lead exposures rather than identifying children who have already been unduly exposed or attempting to ameliorate the toxic effects of lead exposure.

## 5. Conclusions

This CBPR was successful in improving people’s knowledge in identifying sources of lead, complications, and prevention of lead. The involvement of kindergarten students in learning about lead and its prevention using Sesame Street Lead Away videos was exemplary, and easy-to-adopt in other programs. A comprehensive educational strategy addressing multiple groups of people could be the key to success of the project. However, studies are needed to show the effectiveness of health education in improving people’s health behavior. More innovative methods of interventions are needed addressing the needs of high-risk populations and local communities to address their specific health needs for alleviating the risk of lead poisoning in the community.

## Figures and Tables

**Table 1:** Summary of outreach and training provided by Community Lead Awareness Partnership (CLAP) for Healthy Kids.

Activity	Data Collection Procedures	Venue for Events	Training Materials
Provide health education materials, educational training, and presentations to community-based organizations, child-care centers, and faith-based organizations	1. Information, Education, Communication materials given out 2. Sign-in sheets 3. Videos, pictogram, color charts	Churches, Childcare centers, Elementary schools	Sesame Street Lead Away Video [18] (Age of children 3–5). A Teacher’s Guide to How Mother Bear Taught the Children about Lead Source: University of Connecticut. Cooperative Extension System. [19]
Host hands-on training on lead poisoning prevention at local home-builder retail stores	Exit survey	Lowe’s Home Depot Marvin’s Sherwin Williams	Hands-on training and educational materials
Provide lead prevention training for realtors	Follow-up survey	Housing Alternatives of Hattiesburg; Neighborhood Associations	HUD’s Lead-Based Paint Visual Assessment Training [17]
Provide intensive 8 h training on lead safe work practices to inspectors, contractors, and DIY workers	Follow-up survey	City of Hattiesburg Mayor’s Office	PowerPoint presentation; HUD’s Lead-Based Paint Visual Assessment Training [17]
Provide education on lead poisoning prevention through home-buyer education classes	Pre/Post test	Housing Alternatives of Hattiesburg; Neighborhood Associations	PowerPoint presentation
Provide training on lead-safe work practices to rental property owners	Follow-up survey	Churches	PowerPoint presentation

**Table 2:** Impact of the major training activities provided by CLAP for Healthy Kids.

Mode of Training	Participants	Assessment	Outcome Measures
Health fairs	467	-	Distributed 1000 educational materials (leaflets and brochures)
Community events	469	-	Appeared in 25 health events. The City Mayor proclaimed the CLAP for Healthy Kids activities for lead prevention.
Kindergarten classes	241	-	Students offered a certificate of completion
Hands-on training at home-building retail stores	25	Exit survey	23 out of 25 (92%) reported the training was useful or very useful
HUD’s online Lead-Based Paint Visual Assessment Training	220 realtors; 75 inspectors, contractors, and DIY workers	Follow-up survey	59.4%, 67.9%, 65.1% of the realtors identified soil, car batteries, and paint as sources of lead in the environment, respectively
			A total of 62.3%, 48.1%, and 58.5%, at posttest, identified three complications—mental, physical, and psychological, respectively
		Pre- and Post-test	The mean posttest score was significantly higher than the pretest scores (7.47 ± 2.07 *vs*. 6.60 ± 1.68, *p* = 0.04, respectively).
			All the participants at a 2-month follow-up reported that they actually implemented what they had learned during the training on HUD curriculum on lead
Training workshops for home buyers and retail home owners	91	Exit survey	90% mentioned the training was useful or very useful.

## Data Availability

The data presented in this study are available on request from the corresponding author.
